# Leveraging a disulfidptosis-based signature to characterize heterogeneity and optimize treatment in multiple myeloma

**DOI:** 10.3389/fimmu.2025.1559317

**Published:** 2025-04-16

**Authors:** Bingxin Zhang, Dong Zheng, Shuxia Zhu, Xinyi Zhang, Quanqiang Wang, Zhili Lin, Ziwei Zheng, Shujuan Zhou, Zixing Chen, Sisi Zheng, Enqing Lan, Luning Cui, Hansen Ying, Yu Zhang, Xuanru Lin, Qiang Zhuang, Honglan Qian, Xudong Hu, Yan Zhuang, Qianying Zhang, Zhouxiang Jin, Songfu Jiang, Yongyong Ma

**Affiliations:** ^1^ Department of Hematology, The First Affiliated Hospital of Wenzhou Medical University, Wenzhou, Zhejiang, China; ^2^ Department of Hepatobiliary Surgery, The Second Affiliated Hospital and Yuying Children’s Hospital of Wenzhou Medical University, Wenzhou, Zhejiang, China; ^3^ Key Laboratory of Intelligent Treatment and Life Support for Critical Diseases of Zhejiang, Wenzhou, Zhejiang, China; ^4^ Zhejiang Engineering Research Center for Hospital Emergency and Process Digitization, Wenzhou, Zhejiang, China

**Keywords:** multiple myeloma, disulfidptosis, prognostic gene signature, tumor microenvironment, oxidative stress, immunotherapy, targeted drugs

## Abstract

**Background:**

Disulfidptosis is an emerging type of programmed cell death related to ROS accumulation and aberrant disulfide bond formation. Multiple myeloma (MM) is the second most prevalent hematologic malignancy characterized by a high synthesis rate of disulfide bond-rich proteins and chronic oxidative stress. However, the relationship between disulfidptosis and MM is still unclear.

**Methods:**

Using the non-negative matrix factorization and lasso algorithm, we constructed the disulfidptosis-associated subtypes and the prognostic model on the GEO dataset. We further explored genetic mutation mapping, protein-protein interactions, functional enrichment, drug sensitivity, drug prediction, and immune infiltration analysis among subtypes and risk subgroups. To improve the clinical benefits, we combined risk scores and clinical metrics to build a nomogram. Finally, *in vitro* experiments examined the expression patterns of disulfidptosis-related genes (DRGs) in MM.

**Results:**

By cluster analysis, we obtained three subtypes with C2 having a worse prognosis than C3. Consistently, C2 exhibited significantly lower sensitivity to doxorubicin and lenalidomide, as well as a higher propensity for T-cell depletion and a non-responsive state to immunotherapy. Similarly, in the subsequent prognostic model, the high-scoring group had a worse prognosis and a higher probability of T-cell dysfunction, immunotherapy resistance, and cancer cell self-renewal. DRGs and risk genes were widely mutated in cancers. Subtypes and risk subgroups differed in ROS metabolism and the p53 signaling pathway. We further identified eight genes differentially expressed in risk subgroups as drug targets against MM. Then 27 drugs targeting the high-risk group were predicted. Based on the DRGs and risk genes, we constructed the miRNA and TF regulatory networks. The nomogram of combined ISS, age, and risk score showed good predictive performance. qRT-PCR of cell lines and clinical specimens provided further support for prognostic modeling.

**Conclusion:**

Our research reveals the prognostic value of disulfidptosis in MM and provides new perspectives for identifying heterogeneity and therapeutic targets.

## 1 Introduction

Multiple myeloma (MM) is the second most common hematologic malignancy characterized by malignant transformation of plasma cells in the bone marrow, whose uncontrolled growth may lead to hypercalcemia, renal injury, anemia, and destructive bone damage (1). Although the treatment paradigm for MM has made great strides in the past, a cure remains out of reach for most patients. Many patients eventually develop resistance to the standard therapies, which ultimately leads to relapse. The treatment of relapse-refractory MM, especially in patients with risk characteristics, remains a clinical challenge (2). Malignant transformation of plasma cells is frequently accompanied by molecular biological aberrations. The presence of potential molecular drivers leads to heterogeneity in the clinical course (3, 4). The International Staging System (ISS) is based on two simple laboratory indicators: serum albumin and β_2_-microglobulin (β_2_-MG) levels. The revised ISS (R-ISS) further combines high-risk genetic abnormalities, lactate dehydrogenase (LDH) levels, and ISS. The ISS and R-ISS are the most widely used prognostic evaluation systems for MM (5). However, there are still limitations to the heterogeneity and prognostic evaluation of MM patients based on the above staging (6). Therefore, it is imperative to develop reliable and effective prognostic biomarkers to identify high-risk features in MM and to guide customized and optimal treatment.

In addition, there is growing evidence that cancer cells exhibit altered metabolic profiles associated with increased demand for metabolic gene reorganization (7–9). A recent study reported an emerging type of metabolism-related programmed cell death, namely “disulfidptosis” (10). During glucose deprivation, *SLC7A11*-mediated cystine uptake leads to the accumulation of ROS and the formation of abnormal disulfide bonds by consuming intracellular NADPH, thereby promoting cell death by disrupting the conformation of cytoskeletal proteins (10).

Oxidative stress and mutational risk are two important pathogenic mechanisms in MM, and the former can in turn lead to the accumulation of the latter (11, 12). As a tumor with a high protein synthesis and secretion load (13), MM relies on the thioredoxin (Trx) system to reduce endoplasmic reticulum stress and oxidative stress (14). These cells secrete large amounts of immunoglobulins and cytokines requiring the support of rearranged disulfide bonds (13, 15, 16). It relies on the protein disulfide isomerase (PDI) to fold and preserve its structural integrity (17, 18). In addition, actin polymerization is an important process by which myeloma cells home to the BM and interact with its protective microenvironment. However, current studies on the relationship between disulfidptosis and MM remain to be further explored.

In our study, distinct clustering features were identified to explore the heterogeneity of MM based on disulfidptosis-related molecular characteristics. The model associated with disulfidptosis was developed to characterize the immune microenvironment, assess drug sensitivity, and predict the prognosis and immunotherapy sensitivity of MM. Disulfidptosis may provide new avenues for MM risk stratification and metabolic therapy.

## 2 Materials and methods

### 2.1 Data acquisition

The Gene Expression Omnibus (GEO) database (http://www.ncbi.nlm.nih.gov/geo/) was utilized to obtain the gene expression and clinical data of the MM patients (GSE136337, GSE24080, and GSE4204). We normalized the three datasets to increase the comparability between the data. GSE136337 was used to construct tumor subtypes and prognostic models, while the other two were used as validation sets. We screened samples with complete survival information (GSE136337, n = 424; GSE24080, n = 556; GSE4204, n = 534). Subjects with complete clinical data were further identified (GSE136337, n = 415; GSE24080, n = 556). **Table 1** summarizes the included datasets.

**Table 1:** Clinical covariates of the training and validation cohorts.

Characteristics	Training cohort GSE136337 (n = 415)	Validation cohort GSE24080 (n = 556)	Validation cohort GSE4204 (n = 534)
Sex			
Female	158 (38%)	222 (40%)	NA
Male	257 (62%)	334 (60%)	NA
Age			
≤ 65 years	297 (72%)	421(76%)	NA
> 65 years	118 (28%)	135(24%)	NA
Alb			
≥ 3.5 g/dL	331 (80%)	481(87%)	NA
< 3.5 g/dL	84 (20%)	75(13%)	NA
β_2_-MG			
< 3.5 mg/L	187 (45%)	320(58%)	NA
3.5–5.5 mg/L	109 (26%)	118(21%)	NA
≥ 5.5 mg/L	119 (29%)	118(21%)	NA
LDH			
≤ 250 U/L	392 (94%)	507(91%)	NA
> 250 U/L	23 (6%)	49(9%)	NA
del (17p)			
False	400 (96%)	NA	NA
True	15 (4%)	NA	NA
t (4; 14)			
False	401 (97%)	NA	NA
True	14 (3%)	NA	NA
t (14; 16)			
False	414 (99%)	NA	NA
True	1 (1%)	NA	NA
ISS			
I	163 (39%)	296(53%)	NA
II	133 (32%)	142(26%)	NA
III	119 (29%)	118(21%)	NA
R-ISS			
I	149 (36%)	NA	NA
II	243 (59%)	NA	NA
III	23 (5%)	NA	NA
Risk score			
High	206 (50%)	278 (50%)	267 (50%)
Low	209(50%)	278 (50%)	267 (50%)
Survival			
Alive	239 (58%)	386 (69%)	442 (83%)

15 disulfidptosis-related genes (DRGs) include *SLC7A11*, *SLC3A2*, *ABI2*, *BRK1*, *CYFIP1*, *NCKAP1*, *RPN1*, *RAC1*, *WASF2*, *GYS1*, *NDUFS1*, *NUBPL*, *NDUFA11*, *LRPPRC*, and *OXSM* (19).

### 2.2 Gene interaction and the genetic variant landscape

Based on the Pearson correlation coefficients, we used the “circlize” R package to explore the genetic interactions among the DRGs. For proteomics, the STRING database (version 11.5) (20) provides a way to visualize the correlations between the proteins regulated by these genes. Due to limited data on myeloma in The Cancer Genome Atlas, we conducted a pan-cancer analysis of mutations in DRGs and risk genes using Gene Set Cancer Analysis (https://guolab.wchscu.cn/GSCA/#/) (21). The mutation status and loci in cancers were further obtained with the cBioPortal for Cancer Genomics (http://www.cbioportal.org/).

### 2.3 Identification and validation of disulfidptosis-related isoforms

Unsupervised clustering of MM samples is performed using the nonnegative matrix factorization (NMF) clustering algorithm (22). The “lee” function is selected and 500 iterations are performed. The number of clusters k was set from 2 to 6. We choose the optimal rank based on the inflection point at which cophenetic values begin to drop significantly (23–26). The discrimination between the isoforms (C1, C2, and C3) was further validated using principal component analysis (PCA). Since C1 contained a small number of individuals (n = 12), Kaplan-Meier curves were generated to assess the variations in survival between C2 and C3.

### 2.4 Comprehensive analyses of subtypes

To evaluate the variations in medication sensitivity among the clusters, the “pRRophetic” package was utilized. With the “limma” package, differentially expressed genes (DEGs) between C2 and C3 were screened as candidate genes for subsequent prognostic models. To further screen out key DEGs with significant biological significance for constructing prognostic models, we set the threshold criteria as FC > 1.5 and adjusted P < 0.05. Then we explored the biological mechanisms underlying the disulfidptosis-related isoforms via the Kyoto Encyclopedia of Genes and Genomes (KEGG) analysis and Gene Ontology (GO) analysis (P < 0.05, FDR-q < 0.25).

### 2.5 Construction and evaluation of the prognostic model

Prognosis-associated DEGs were further identified by Cox regression analysis to serve as candidate genes for prognostic model construction (P < 0.05). Then, we used the “glmnet” package (27) and conducted 10-fold cross-validation to construct a lasso Cox regression prognostic model based on lambda. min. Lambda.min directly corresponds to the minimum of the cross-validation error under preserving more features, and it is chosen to provide more accurate prediction results and help identify potential biomarkers. Depending on the median score, the individuals were classified into low- and high-risk subgroups. To evaluate the prognostic prediction effectiveness, the area under the curve (AUC) was computed using the “timeROC” package.

### 2.6 Exploration of biological functions and drug targets based on the model

In the differential analysis among the risk groups, we set lower thresholds (FC > 1.2, adjusted P < 0.05) for comprehensive exploration of gene expression changes, and performed KEGG and Gene Set Enrichment Analysis (GSEA v4.2.2 software, P < 0.05 and FDR-q < 0.25) to explore potential biological functions.

We subsequently explored the genes targeted by MM drugs among DEGs between risk subgroups through the DrugBank database (https://go.drugbank.com/) (28). Furthermore, to identify more effective treatments targeting the high-risk group, we employed human cancer cell line gene expression data from the Cancer Cell Line Encyclopedia (https://depmap.org/portal/) as a training set and calculated dose-response AUC to quantify the drug sensitivity of the high-risk subgroup by using the “pRRophetic” package. The AUC was negatively associated with drug responsibility. The Cancer Therapeutics Response Portal (CTRP) (https://portals.broadinstitute.org/ctrp.v2.1/) (29) and PRISM Repurposing (https://depmap.org/portal/prism/) (30) are sources of drug sensitivity data. Next, we conducted differential drug response analysis between the highest and lowest risk score deciles (log_2_FC > 0.1). Then compounds that showed a negative correlation with the risk score were examined (Spearman’s r < -0.2) (31).

### 2.7 Analysis of miRNA and transcription factors

Based on miRWalk database (current version: January 2022) (http://129.206.7.150/), we searched in the “Disease” column (DOID: 9538#multiple myeloma) and finally obtained miRNAs related to MM. Then we intersected them with DEGs among clusters and risk subgroups. In addition, we inputted DRGs and prognostic genes into the Cistrome DB database (http://dbtoolkit.cistrome.org/) (Default parameters) and obtained transcription factors (TFs) that regulate these genes. The miRNA and TF regulatory networks were finally visualized by Cytoscape (v3.9.1).

### 2.8 Characterization of the immunophenotype and immunotherapy responsiveness among subgroups

The number of immune cells in the tumor microenvironment across subtypes was determined using the single-sample gene set enrichment analysis (ssGSEA) and the xCell (32). Tumor immune dysfunction and exclusion (TIDE) was performed to analyze the function and infiltration level of T cells (33). Both TIDE (33) and immunophenotype score (IPS) (34) can assess the sensitivity of immune checkpoint blockade (ICB). IPS addresses the immunological phenotype of the cells from four perspectives (“antigen-presenting (AP), effector cells (EC), suppressor cells (SC), and checkpoints (CP)”). The indicator balance resulted in the generation of a total score (z-score, AZ). Greater z-scores and lower TIDE were associated with increased immunotherapy responsiveness (33, 34). Moreover, we conducted a correlation analysis between prognostic genes and immune-related genes, pathways, and functions (35). To assess the self-renewal ability of the samples, the mRNA expression-based stemness index (mRNAsi) was calculated according to transcriptomic and epigenetic characteristics (36).

### 2.9 Establishing a predictive nomogram

Through univariate and multivariate Cox regression, we screened for independent prognostic factors to be included in the construction of a nomogram. The construction of the nomogram is achieved through the “rms” package. For the internal validation, a calibration curve was developed. Time-ROC curves for 1-, 3- and 5-year survival compared between the nomogram and other factors (37).

### 2.10 Cell lines and cell culture

Cells for RPMI8226, MM1.R, U266, NCI-H929, and LP-1 were purchased from Fenghui Biotechnology Co., Ltd (Hunan, China). The cells were grown in RPMI-1640 media (Gibco, Shanghai, China) containing 10% fetal bovine serum, 0.1 mg/ml streptomycin, and 100 U/ml penicillin G. The medium was then incubated with the cells at 37°C and 5% CO_2_ in a humid environment.

### 2.11 Patients

50 MM patients from the First Affiliated Hospital of Wenzhou Medical University were included in the study. Additionally, BM from 24 healthy donors served as a control group for cell lines and patient samples. The clinical characteristics of the patients are shown in **Table 2**. All samples were taken with the subjects’ informed consent. This research was approved by the Ethics Committee in Clinical Research of the First Affiliated Hospital of Wenzhou Medical University and followed the Declaration of Helsinki.

**Table 2:** The clinical features of the subjects included in this experiment.

Variables	Levels	MM (n = 50)	Normal (n = 24)	P
Sex	Female	18 (36%)	10 42%)	0.638
	Male	32 (64%)	14 (58%)	–
Age (years)	< 65	14 (28%)	9 (38%)	0.408
	≥ 65	36 (72%)	15 (62%)	–
Isotype	IgG	27 (54%)	–	–
	IgA	13 (26%)	–	–
	IgD	1 (2%)	–	–
	IgM	1 (2%)	–	–
	Light chain κ	5 (10%)	–	–
	Light chain λ	3 (6%)	–	–
Albumin (g/dL)	≥ 3.5	20 (40%)	–	–
	< 3.5	30 (60%)	–	–
β_2_-MG (mg/L)	< 3.5	17 (34%)	–	–
	3.5–5.5	12 (24%)	–	–
	≥ 5.5	21 (42%)	–	–
LDH (U/L)	≤ 250	41 (82%)	–	–
	> 250	9 (18%)	–	–
del (17p)	False	49 (98%)	–	–
	True	1 (2%)	–	–
IgH rearrangement	False	46 (92%)	–	–
	True	4 (8%)	–	–
del (13q)	False	37 (74%)	–	–
	True	13 (26%)	–	–
amp 1q	False	37 (74%)	–	–
	True	13 (26%)	–	–
ISS	I	9 (18%)	–	–
	II	20 (40%)	–	–
	III	21 (42%)	–	–
R-ISS	I	8 (16%)	–	–
	II	37 (74%)	–	–
	III	5 (10%)	–	–
Myeloma cells (%)	< 10	18 (36%)	–	–
	≥ 10	32 (64%)	–	–
Calcium (mmol/L)	≤ 2.65	44 (88%)	–	–
	> 2.65	6 (12%)	–	–
Serum creatinine (μmol/L)	< 177	41 (82%)	–	–
	≥ 177	9 (18%)	–	–
Hb (g/L)	≥ 85	23 (46%)	–	–
	< 85	27 (54%)	–	–
Bone lesions	0	18 (36%)	–	–
	1-3	4 (8%)	–	–
	> 3	28 (56%)	–	–

### 2.12 RNA extraction, reverse transcription, and quantitative real-time PCR

RNA was extracted from bone marrow samples by Righton DNA&RNA Blood and Tissue Kit (Righton Bio, Shanghai, China), followed by reverse transcription with the cDNA Synthesis Kit (Vazyme, Nanjing, China). Finally, quantitative PCR was performed using Taq Pro Universal SYBR qPCR Master Mix (Vazyme, Nanjing, China). Internal controls were implemented using *β-ACTIN*. The comparative threshold cycle (Ct) approach was used to determine relative expression (38). The primer sequences used are as follows:

*CR2* forward primer: 5′‐AAAGGGCTGGAACCAAGGAA‐3′;
*CR2* reverse primer: 5′‐GACAGGAGCAAGTGAACGGGA‐3′;
*DIRAS3* forward primer: 5′‐CTGCCGACCATTGAAAATACCT‐3′;
*DIRAS3* reverse primer: 5′‐GACTGAGTAGACCAGGACGAAGG‐3′;
*FOSB* forward primer: 5′‐GAGACAGATCAGTTGGAGGAAGAA‐3′;
*FOSB* reverse primer: 5′‐CACAAACTCCAGACGTTCCTTC‐3′;
*GJB2* forward primer: 5′‐GAGTGAATTTAAGGACATCGAGGAG‐3′;
*GJB2* reverse primer: 5′‐TGCATGGAGAAGCCGTCGTA-3′;
*HK2* forward primer: 5′‐TTGGAGCCACCACTCACCCTA‐3′;
*HK2* reverse primer: 5′‐GAGCCCATTGTCCGTTACTTTC-3′;
*KIF21B* forward primer: 5′‐GTCAAGGTGGCCGTCAGGAT‐3′;
*KIF21B* reverse primer: 5′‐TTCTTGCCAGGTGTCCAGGTC-3′;
*LY6E* forward primer: 5′‐AATCTGTACTGCCTGAAGCCGA‐3′;
*LY6E* reverse primer: 5′‐CCAAATGTCACGAGATTCCCA-3′;
*PLTP* forward primer: 5′‐GCTGGCTCTGATCCCATTACAG‐3′;
*PLTP* reverse primer: 5′‐AATCCCGCATGGTTCGTCA-3′;
*SHROOM3* forward primer: 5′‐CTCACGGACATCAAGCTCAACAA‐3′;
*SHROOM3* reverse primer: 5′‐CCTTTCTTCATTACTGGCATCTTCA-3′;
*TEAD1* forward primer: 5′‐CCAACCATTCTTACAGTGACCCAT‐3′;
*TEAD1* reverse primer: 5′‐TCAAACCTTGCATACTCCGTCTC-3′;
*β-ACTIN* forward primer: 5′‐TCAAGATCATTGCTCCTCCTGAG‐3′;
*β-ACTIN* reverse primer: 5′‐ACATCTGCTGGAAGGTGGACA‐3′.

### 2.13 Statistical analyses

R 4.1.1, SPSS 24.0, and GraphPad Prism 9.0.0 were employed in statistical analyses. For comparison of differences, Student’s t-test and ANOVA were used for normal data, while the Wilcoxon test corresponded to skewed data. Multiple comparisons use FDR to correct P-values. As for correlation analysis, Pearson correlation was applied to the bivariate normal distribution, otherwise Spearman rank analysis was used. Survival analysis was performed using the Kaplan-Meier method and the log-rank test was used to compare survival probabilities.

## 3 Results

### 3.1 Gene interaction and the genetic variant landscape

**Figure 1:**
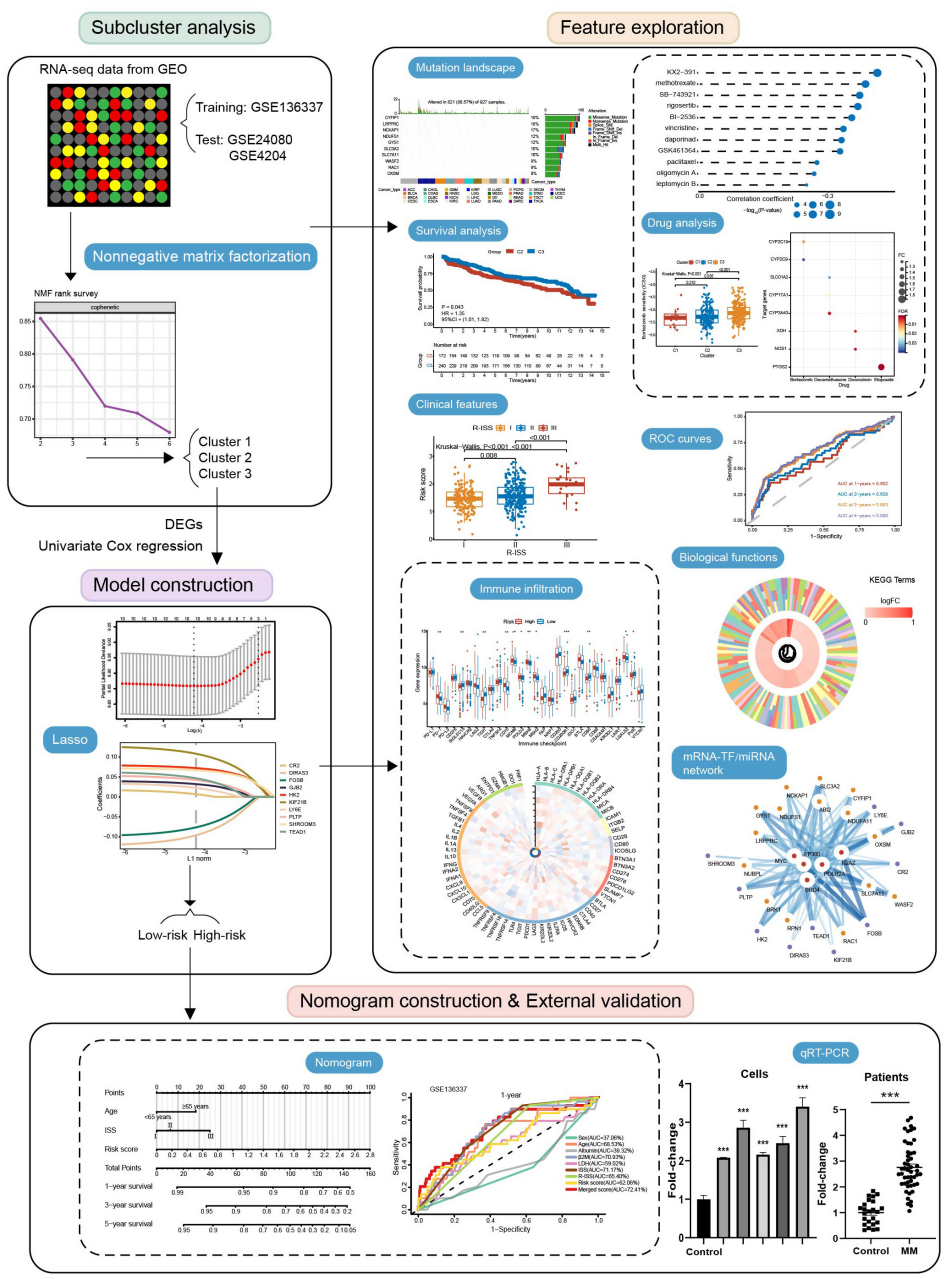
Flow chart of the study.

**Figure 2:**
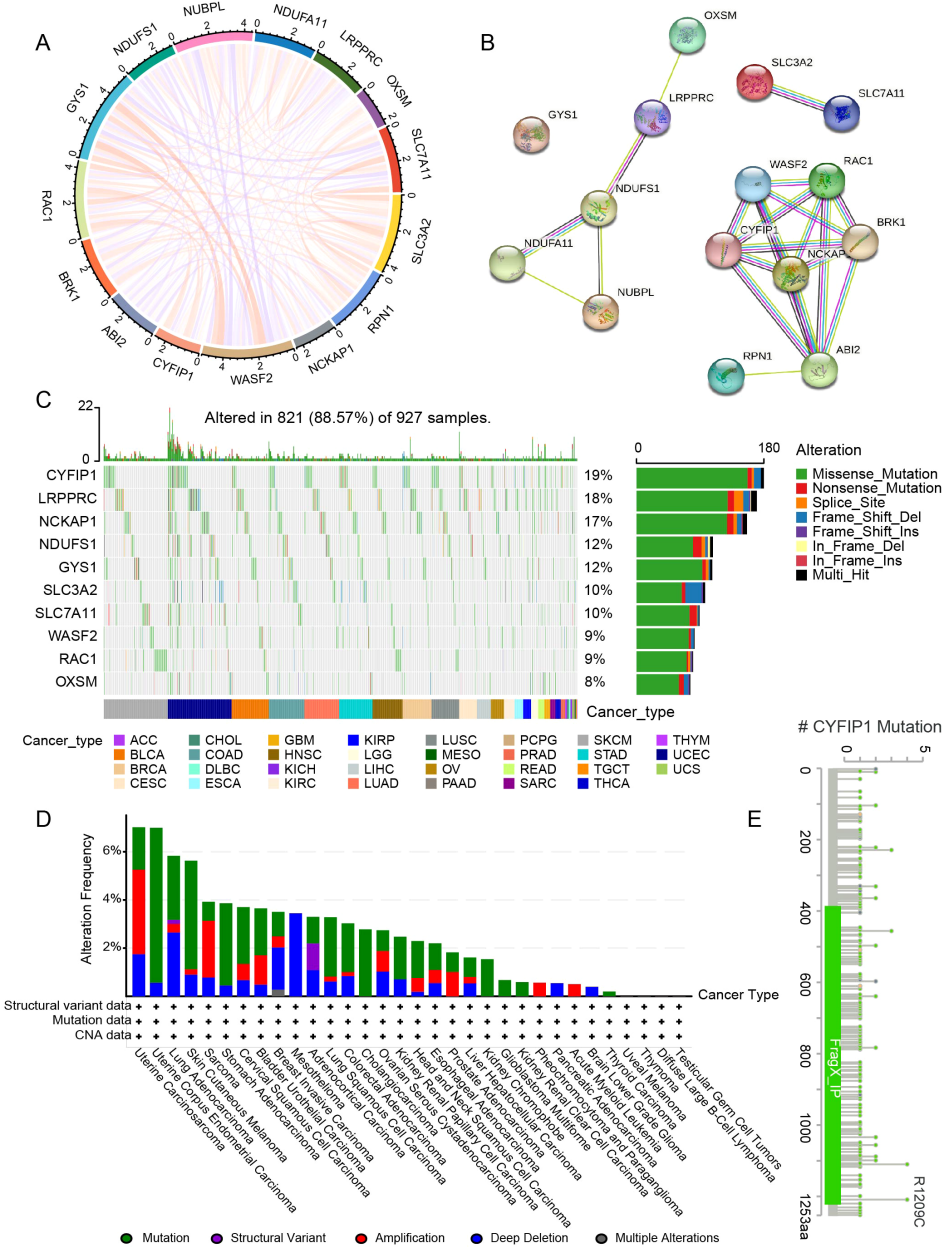
Gene interactions and the genetic variant landscape of DRGs. **(A)** The correlation network of DRGs. **(B)** The protein-protein interaction network of DRGs. **(C)** Pan-cancer analysis of the mutation landscape of DRGs. **(D)** The mutation status of *CYFIP1* in cancers from the cBioPortal. **(E)** Major mutation sites of *CYFIP1*.

The study design is presented in **Figure 1**. These genes were closely related to each other (**Figures 2A, B**). In addition, these genes exhibited widespread mutations in cancer. *CYFIP1* had the highest mutation rate (19%), followed by *LRPPRC* (18%) and *NCKAP1* (17%) (**Figure 2C**). Moreover, missense mutations were the most common type of mutation (**Figure 2C**). The cBioPortal analysis further validated the extensive mutation status of *CYFIP1* in multiple cancers (**Figure 2D**). **Figure 2E** demonstrated the mutation site of *CYFIP1*.

### 3.2 Identification and validation of disulfidptosis-related isoforms

**Figure 3:**
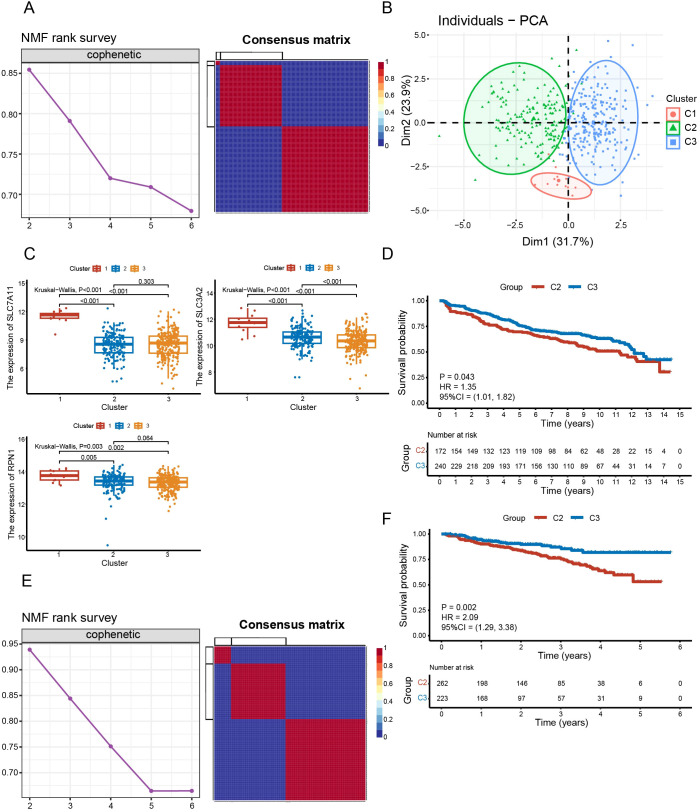
Identification and validation of disulfidptosis-related isoforms. **(A)** The cophenetic curve of NMF clustering at ranks = 2 to 6 and the consensus matrix when rank = 3 in the training dataset. **(B)** Principal component analysis of three clusters. **(C)** Comparison of expression levels of *SLC7A11*, *SLC3A2* and *RPN1* between subgroups. **(D)** Survival analysis between C2 and C3 in the training set (P = 0.043). **(E)** The cophenetic curve of NMF clustering at rank = 2 to 6 and the consensus matrix when rank = 3 in validation dataset GSE4204. **(F)** Survival analysis between C2 and C3 in the validation set GSE4204 (P = 0.002).

The training set was clustered using the NMF algorithm. The decrease of cophenetic value is obviously slowed down after rank=3 (**Figure 3A**). And there is a clear block structure in the consensus matrix at rank=3 (**Figure 3A**), indicating a high stability of the clustering results. The accuracy and stability of this clustering analysis was further demonstrated by PCA (**Figure 3B**). We selected the top three suppressor hits, *SLC7A11*, *SLC3A2* and *RPN1*, from the CRISPR-Cas9 screening in Liu et al.’s study (10). Differential analysis revealed that the expression levels of these three genes in C1 were higher than those in C2 and C3 (P < 0.01) (**Figure 3C**). And the expression of *SLC3A2* in C2 was higher than that in C3 (P < 0.001) (**Figure 3C**). This suggests that they are more susceptible to disulfidptosis. Furthermore, Kaplan-Meier curve revealed that C2 had worse survival than C3 (P = 0.043) (**Figure 3D**). Similar results were replicated in the GSE4204 validation set (P = 0.002) (**Figures 3E, F**).

### 3.3 Comprehensive analyses of subtypes

**Figure 4:**
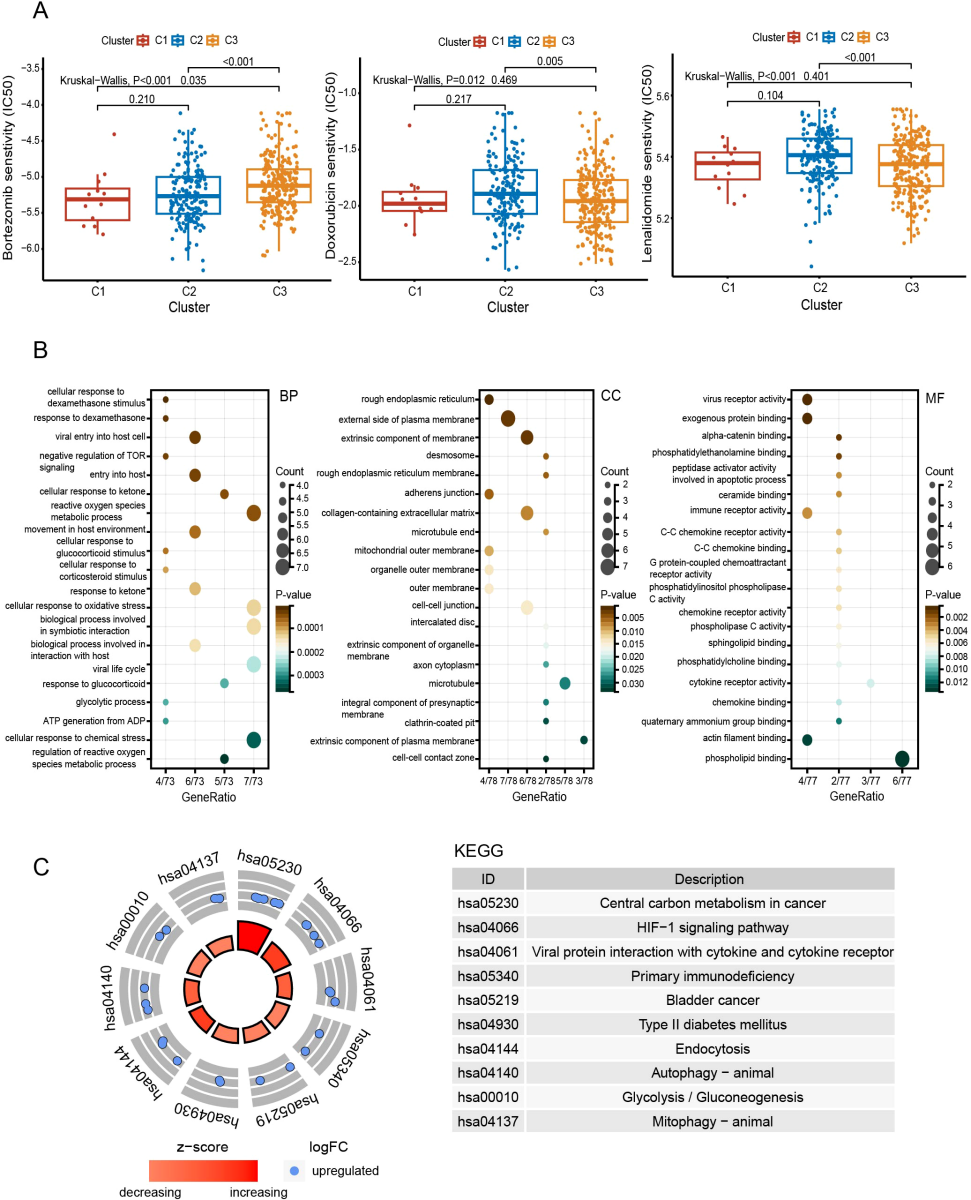
Comprehensive analyses of subtypes. **(A)** Comparison of drug sensitivity between subgroups. **(B)** Functional enrichment analysis with GO. **(C)** KEGG pathway enrichment analysis. BP, biological process; CC, cellular component; MF, molecular function.

Additionally, we found heterogeneity in drug responsiveness between the two subtypes (**Figure 4A**). C1 and C2 were more sensitive to bortezomib than C3 (P < 0.05) (**Figure 4A**). While on doxorubicin and lenalidomide, C3 was more sensitive than C2 (P < 0.01) (**Figure 4A**).

Compared to C3, the pathways enriched in C2 include the HIF-1 signaling pathway, autophagy, glycolysis, actin filament binding, and cellular responses to dexamethasone and oxidative stress (**Figures 4B, C**).

**Figure 5:**
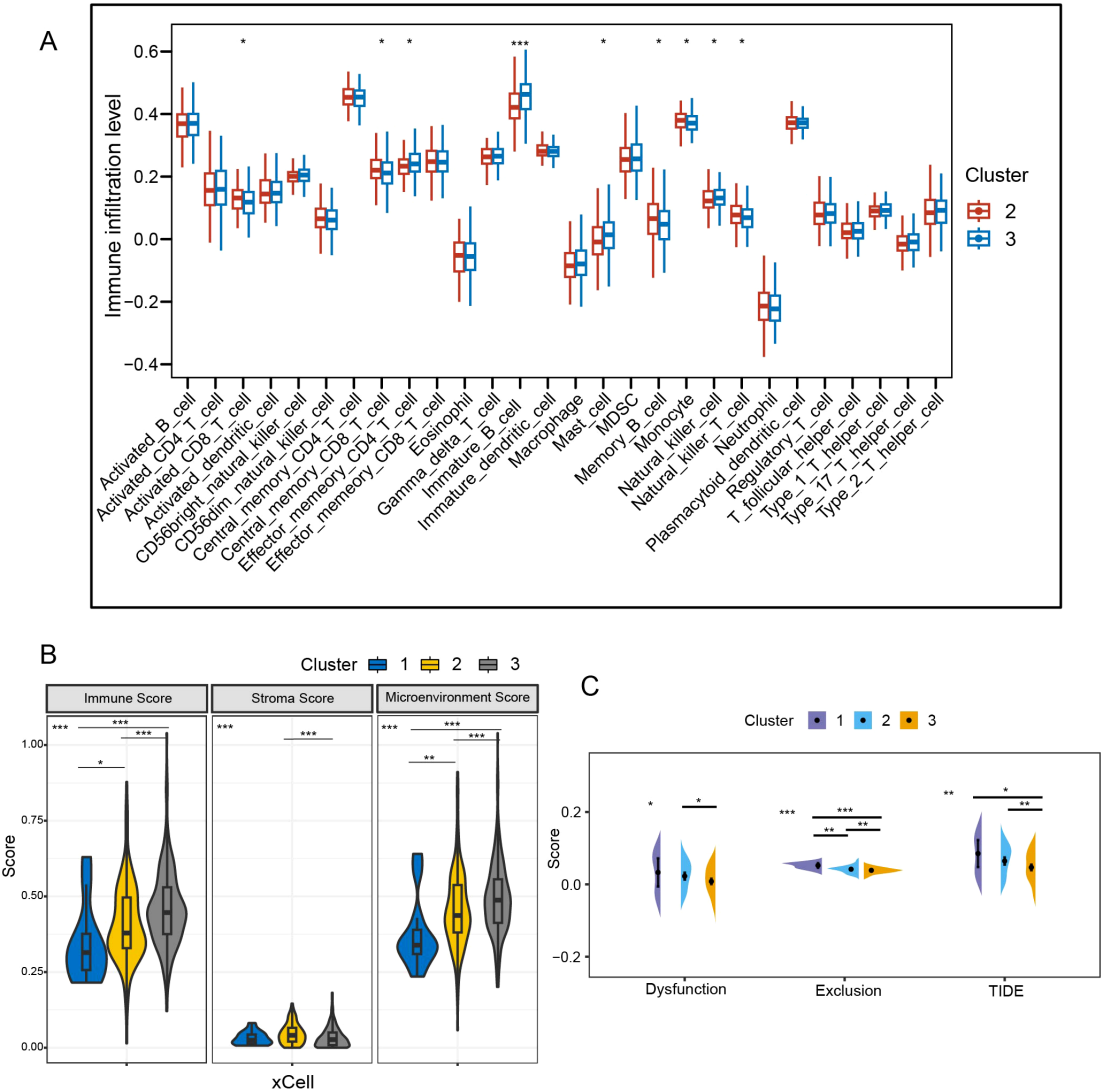
Comparative analysis of immune background between clusters. **(A)** Analysis of intersubtype immune cell infiltration with ssGSEA. **(B)** Immunological comparison between subtypes with xCell algorithm. **(C)** Assessment of T-cell dysfunction and exclusion. TIDE, tumor immune dysfunction and exclusion; *P < 0.05; **P < 0.01; ***P < 0.001.

C2 had higher levels of activated CD8^+^ T cells and central memory CD8^+^ T cells (P < 0.05), while C3 was enriched in effector memory CD4^+^ T cells and NK cells (P < 0.05) (**Figure 5A**). In the xCell algorithm, C1 showed the lowest immunity score, followed by C2 (P<0.05) (**Figure 5B**). However, the stroma score of C2 was higher than that of C3 (P<0.001). Further analysis of T cells revealed that the T-cell exclusion was highest in C1, followed by C2 (P < 0.01) (**Figure 5C**). Additionally, C2 was more prone to T-cell dysfunction (P < 0.05). The TIDE of C1 and C2 was higher than that of C3 (P < 0.05), indicating a greater possibility of immune escape and resistance to immunotherapy in C1 and C2.

### 3.4 Construction and evaluation of the prognostic model

**Figure 6:**
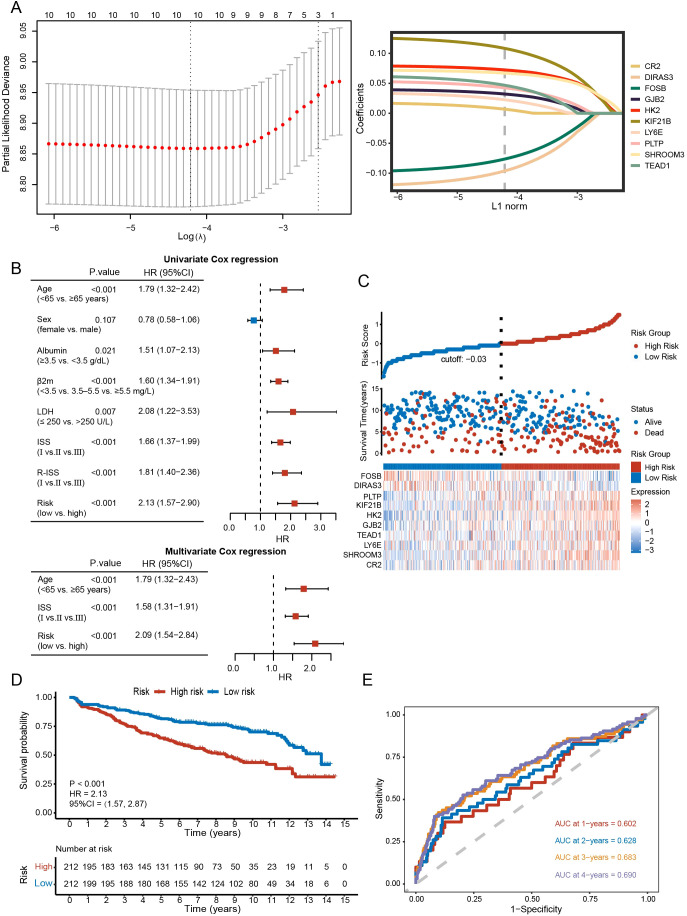
Construction and evaluation of the prognostic model. **(A)** Prognostic model constructed with lasso algorithm. **(B)** Univariate and multivariate analysis. **(C)** Distribution of survival status between high- and low-risk groups and gene expression heatmap of the prognostic model. **(D)** Kaplan-Meier curve of high- and low-risk groups (P < 0.001). **(E)** The sensitivity and specificity of the model were assessed by time-ROC analysis.

We further included 90 DEGs in Cox regression analysis (P<0.05) and ultimately obtained 10 candidate genes. Using the lasso algorithm, a prognostic model related to disulfidptosis was constructed as follows (λ = 0.015) (**Figure 6A**): risk score = (0.0068 × *CR2*) + (0.0670 × *SHROOM3*) + (0.0226 × *LY6E*) + (0.0468 × *TEAD1*) + (0.0322 × *GJB2*) + (0.0724 × *HK2*) + (0.1084 × *KIF21B*) + (0.0425 × *PLTP*) - (0.0961 × *DIRAS3*) - (0.0763 × *FOSB*). The sample was separated into high- and low-risk categories according to median scores. The risk score, age, and ISS proved to be independent prognostic factors (**Figure 6B**).

**Figure 6C** and **Supplementary Figure 1A** revealed differences in survival and gene expression between the high- and low-risk subgroups. In both the training and validation sets, higher scorers fared worse in terms of survival (GSE136337: HR = 2.13, 95% CI = 1.57-2.87, P < 0.001; GSE24080: HR = 1.76, 95% CI = 1.30-2.40, P < 0.001; GSE4204: HR = 1.76, 95% CI = 1.16-2.67, P = 0.008) (**Figure 6D**; **Supplementary Figure 1B**). In the training set, the Brier scores for 1-, 2-, 3-, and 4-year survival of the model were 0.050, 0.057, 0.102, and 0.129, respectively. This further validates the stability of the model. AUCs of the 1-, 2-, 3-, and 4-year survival were 0.602, 0.628, 0.683, and 0.690, respectively, in the GSE136337 (**Figure 6E**), 0.631, 0.682, 0.710, and 0.648 in the GSE24080, 0.663, 0.707, 0.697, and 0.648 in the GSE4204 (**Supplementary Figure 1C**).

### 3.5 Construction and evaluation of the prognostic model

**Figure 7:**
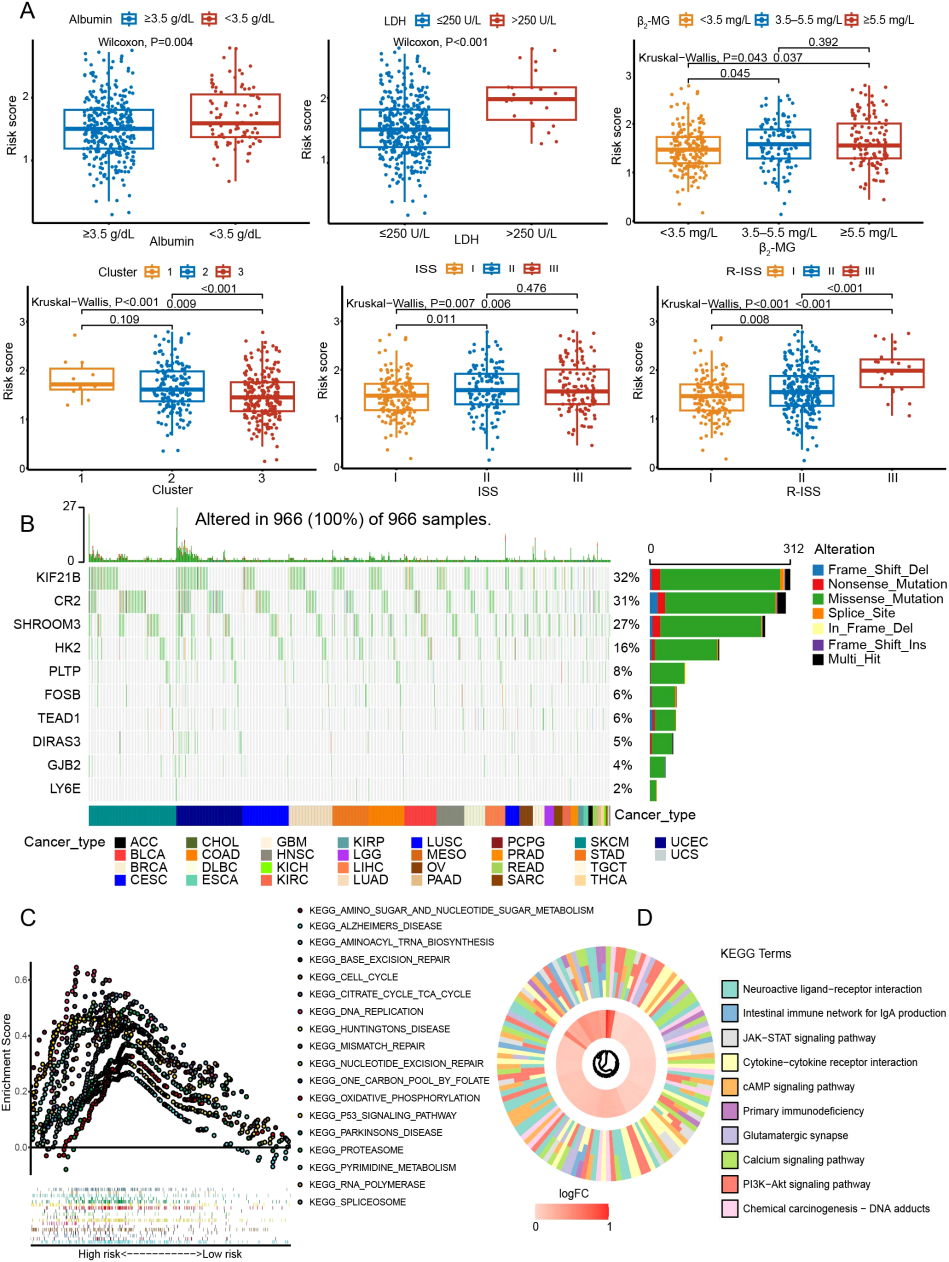
Exploration of the mutation landscape and pathways in the prognostic model. **(A)** Relationships between the risk score and various clinical characteristics. **(B)** The mutation landscape of genes in the model. **(C)** Exploration of biological functions with GSEA. **(D)** KEGG pathway enrichment analysis.

The risk subgroups differed in clinical risk indicators (**Figure 7A**). Those with high levels of LDH and β_2_-MG had higher risk scores, in contrast to albumin (P < 0.05). Higher ISS or R-ISS staging was more concentrated in the high-rated group (P < 0.05). Consistently, C2 with a worse prognosis favored higher risk scores (P < 0.05). In the genetic landscape, *KIF21B* (32%), *CR2* (31%), and *SHROOM3* (27%) were the three genes with the highest mutation rates (**Figure 7B**). Based on the 1215 DEGs, we found that high-risk groups were enriched in pathways including p53 signaling pathway, proteasome, TCA cycle, oxidative phosphorylation, PI3K-Akt signaling pathway, chemical carcinogenesis, and JAK-STAT signaling pathway (**Figures 7C, D**).

### 3.6 Comparative analysis of immune background between risk subgroups

**Figure 8:**
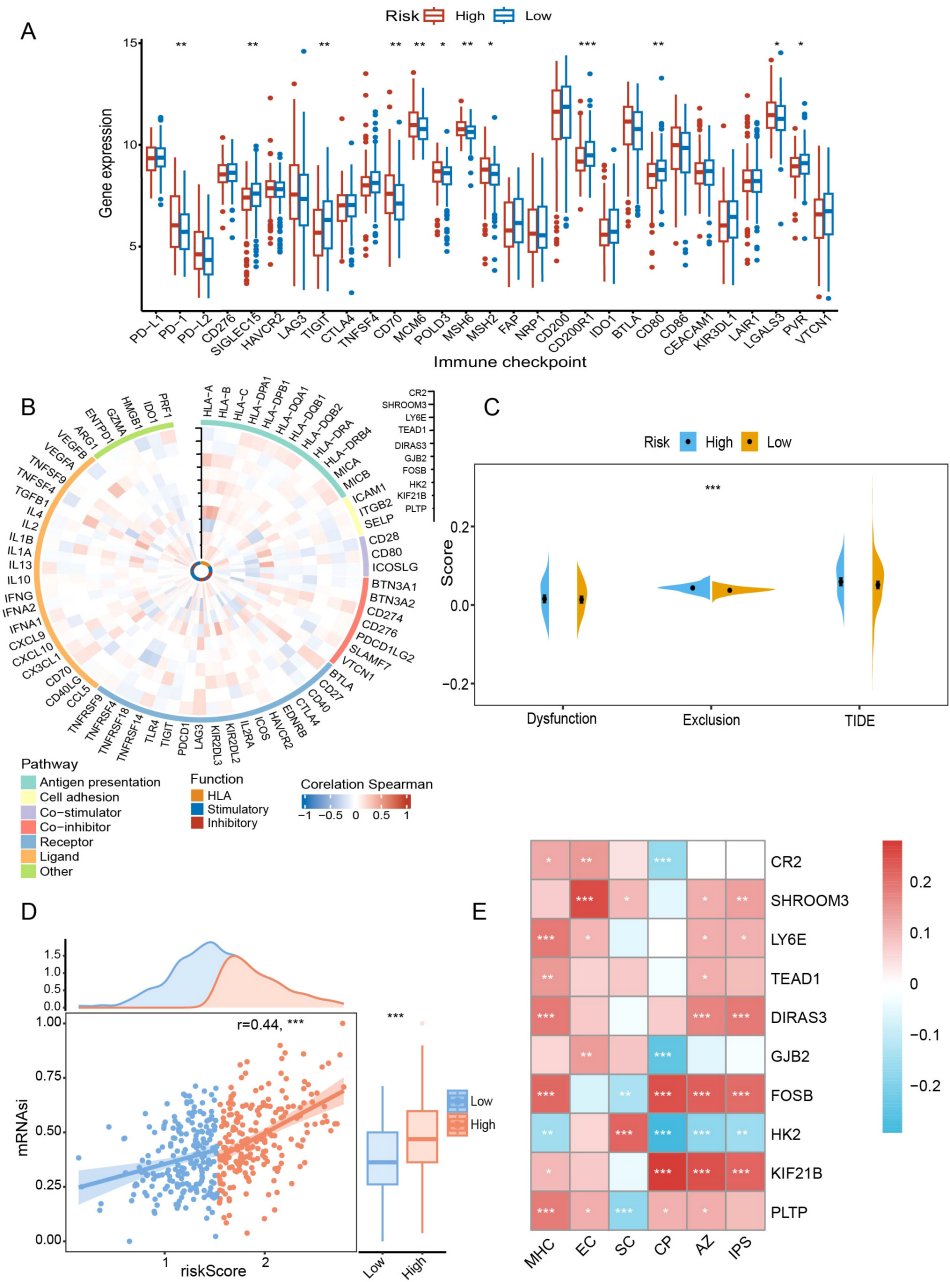
Comparative analysis of immune background between risk subgroups. **(A)** Comparison of immune checkpoints between risk subgroups. **(B)** Correlations of prognostic genes with immune-related pathways and functions. **(C)** Assessment of T-cell dysfunction and exclusion. **(D)** Correlation of risk scores with mRNAsi scores. **(E)** Association of prognostic genes with IPS-related scores. mRNAsi, mRNA expression-based stemness index; TIDE, tumor immune dysfunction and exclusion; IPS, immunophenotype score; MHC, antigen presentation; EC, effector cells; SC, suppressor cells; CP, checkpoint marker; AZ, z-score. *P < 0.05; **P < 0.01; ***P < 0.001.

In terms of the immune checkpoint, the high-risk group had higher expression levels of *PD-1*, *CD70*, *MCM6*, *POLD3*, *MSH6*, *MSH2*, and *LGALS3* (P < 0.05) (**Figure 8A**). For immunity, prognostic genes were closely associated with immune-related pathways and functional status (**Figure 8B**). For example, the expression of suppressive immune genes was generally favorably connected with *GJB2*, whereas the stimulatory immune genes were negatively correlated. Furthermore, T cells in the high-risk group were more susceptible to exclusion (P < 0.001) (**Figure 8C**). In addition, we discovered a positive correlation (r = 0.44, P < 0.001) between mRNAsi and the risk score (**Figure 8D**). A greater chance of recurrence was evident in the high-scoring group.

In subsequent immunophenotypic analyses, *DIRAS3* and *FOSB* were positively correlated with antigen presentation-associated markers (P < 0.001), whereas *HK2* was negatively correlated (P < 0.01) (**Figure 8E**). Similarly, in terms of inhibitory cell-associated features, *FOSB* showed a negative correlation (P < 0.01), whereas the opposite was true for *HK2* and *SHROOM3* (P < 0.05) (**Figure 8E**). Finally, *DIRAS3* and *FOSB* correlated with higher total scores (P < 0.001) (**Figure 8E**), which was associated with greater ICB responsiveness.

### 3.7 Targeted drug prediction and the network of mRNAs and interrelated miRNAs, TFs

**Figure 9:**
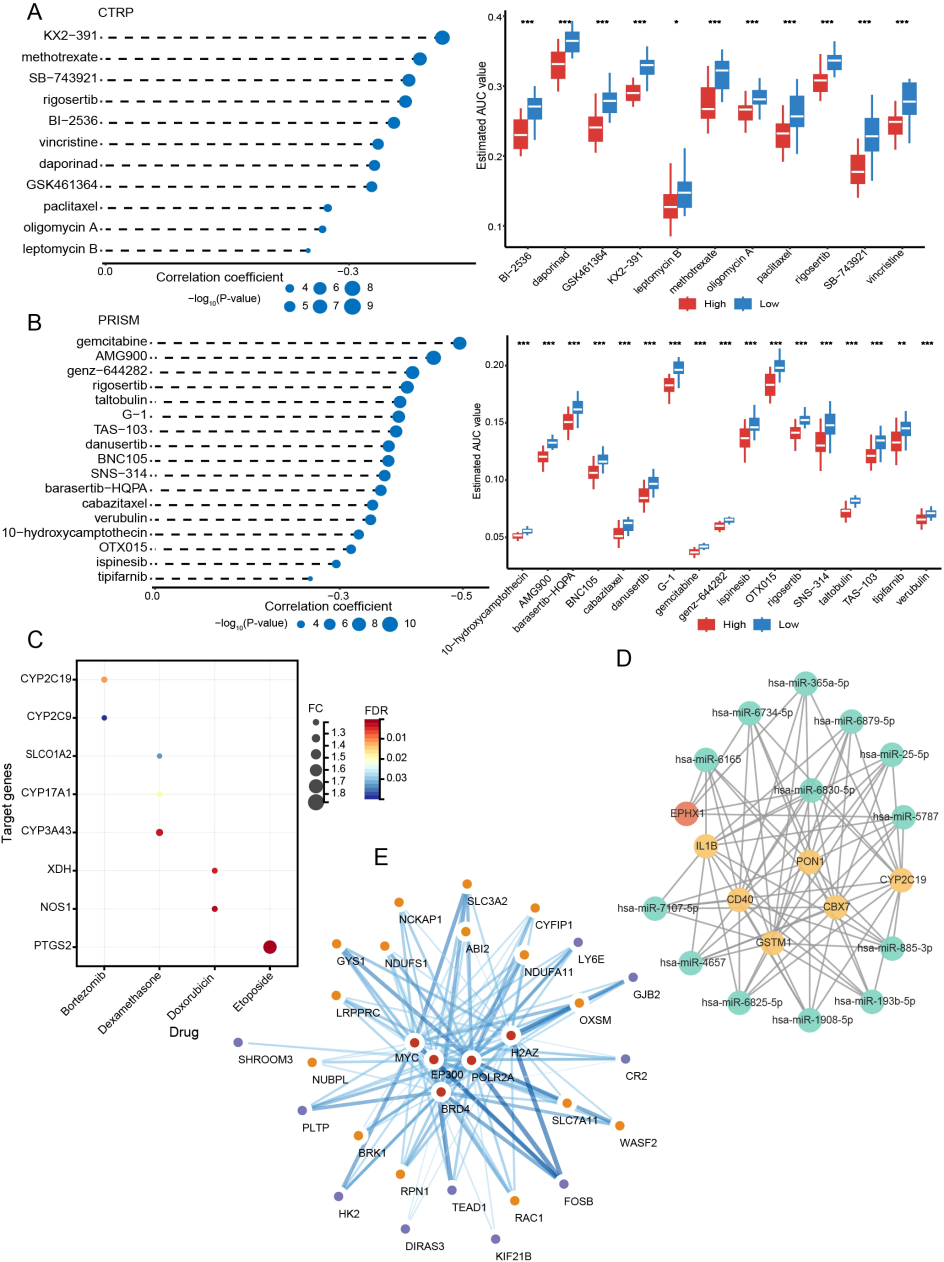
Targeted drug prediction and the network of mRNAs and interrelated miRNAs, TFs. **(A)** The results of correlation analysis and differential drug response analysis in CTRP. **(B)** The results of correlation analysis and differential drug response analysis in PRISM. **(C)** Drug target analysis. **(D)** mRNA-miRNA network. **(E)** mRNA-TF network. *P < 0.05; **P < 0.01; ***P < 0.001.

A total of 27 compounds were predicted to potentially target people in high-risk groups (CTRP: paclitaxel, methotrexate, BI-2536, oligomycin A, daporinad, vincristine, GSK461364, leptomycin B, SB-743921, rigosertib, KX2-391; PRISM: cabazitaxel, danusertib, TAS-103, gemcitabine, BNC105, AMG900, verubulin, OTX015, rigosertib, G-1, tipifarnib, 10-hydroxycamptothecin, barasertib-HQPA, ispinesib, SNS-314, taltobulin, genz-644282). All compounds had lower AUCs in the high-scoring subgroup (P < 0.05) (**Figures 9A, B**). In addition, we further identified eight genes among DEGs in risk subgroups as targets of drugs commonly used in MM through the DrugBank database: *CYP2C19*, *CYP2C9*, *SLCO1A2*, *CYP17A1*, *CYP3A43*, *XDH*, *NOS1*, and *PTGS2* (**Figure 9C**).

Using the miRWalk database, we predicted 13 miRNAs that were closely interlinked with DEGs between subtypes and risk subgroups (miR-365a-5p, miR-6734-5p, miR-6165, miR-6879-5p, miR-25-5p, miR-6830-5p, miR-5787, miR-7107-5p, miR-4657, miR-6825-5p, miR-1908-5p, miR-193b-5p, miR-885-3p) (**Figure 9D**). With the Cistrome DB database, five possible TFs (BRD4, EP300, MYC, POLR2A, and H2AZ) may regulate the expression of DRGs and prognostic risk genes (**Figure 9E**).

### 3.8 Establishing a predictive nomogram

**Figure 10:**
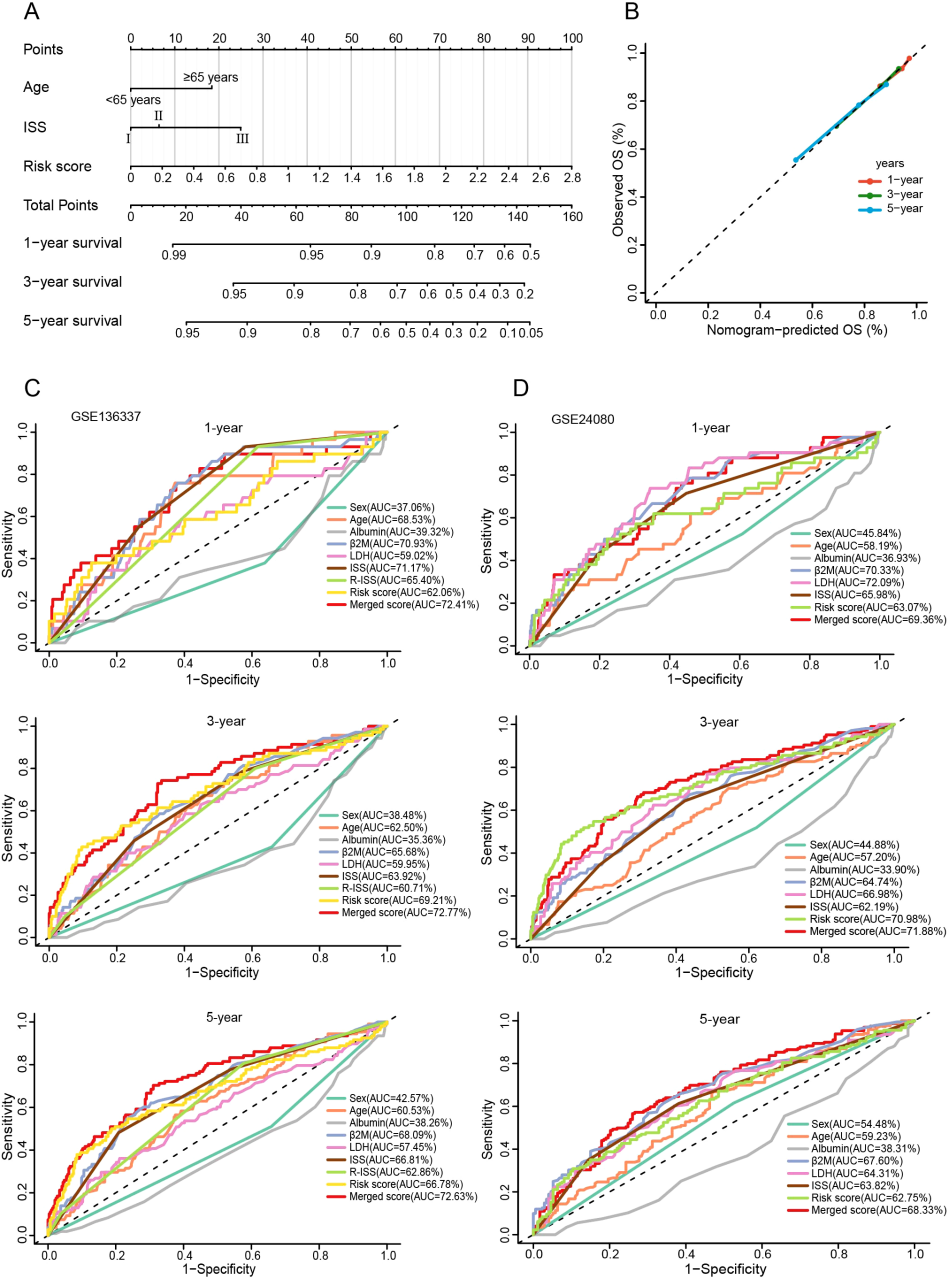
Construction and evaluation of the nomogram. **(A)** The nomogram assembling age, ISS, and risk score. **(B)** Calibration curve of the nomogram. **(C, D)** Time-ROC curves for 1-, 3-, and 5-year survival predictions for the nomograms compared with other clinical traits.

Nomograms can visualize complex regression equations and facilitate the prediction of the probability of an individual’s outcome in medical research and clinical practice. Based on existing data, we constructed a nomogram by combining age, ISS, and the risk score (**Figure 10A**). Self-validation was obtained in the calibration curve (**Figure 10B**). The 1-, 3-, and 5-year AUCs exceeded those of the ISS and R-ISS (GSE136337: 72.41%, 72.77%, and 72.63%; GSE24080: 69.36%, 71.88%, and 68.33%) (**Figures 10C, D**).

### 3.9 External validation with experiments

**Figure 11:**
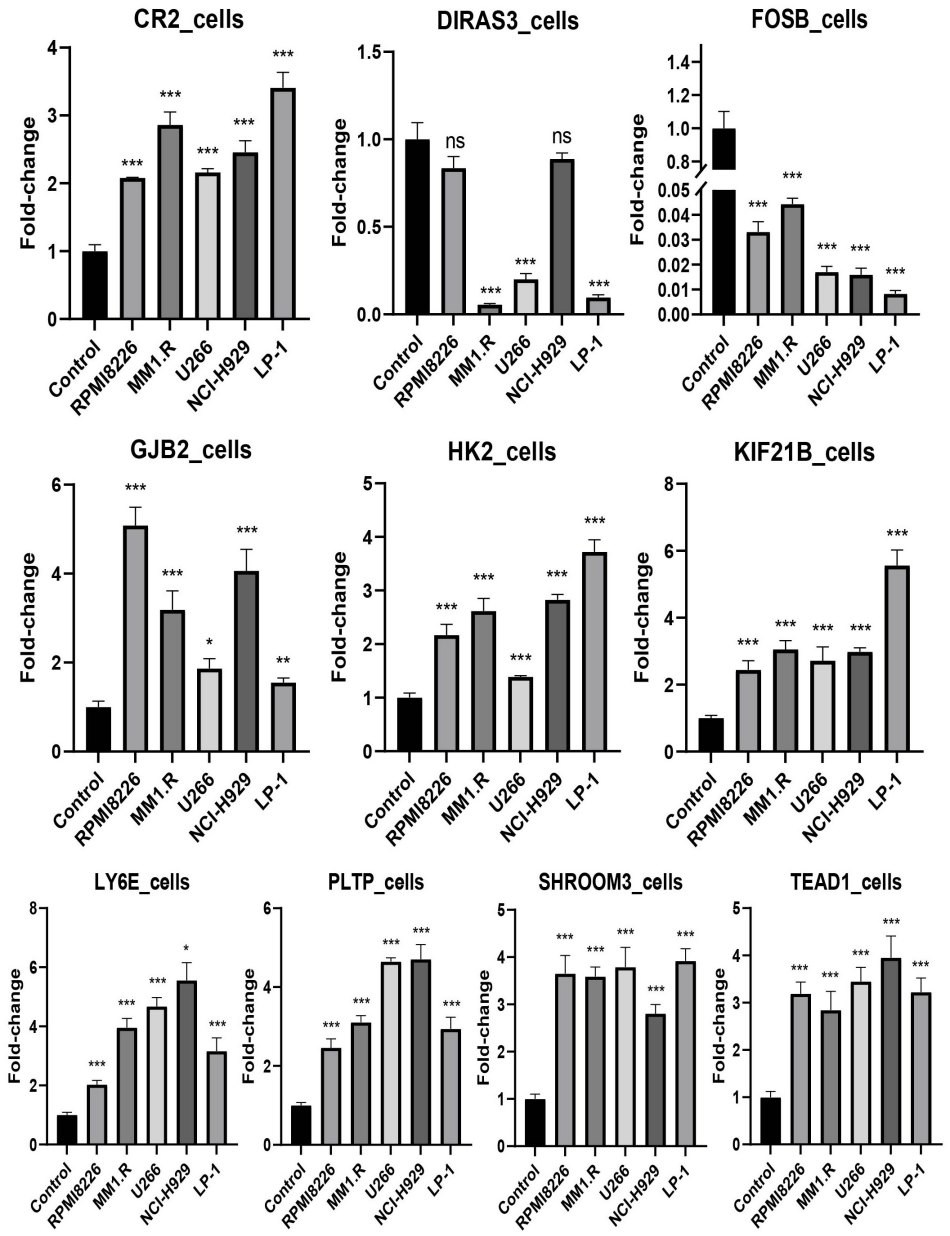
Experimental validation in cell lines (mean ± SEM). ns, no significance; *P < 0.05; **P < 0.01; ***P < 0.001. The experiments were independently repeated 3 times.

**Figure 12:**
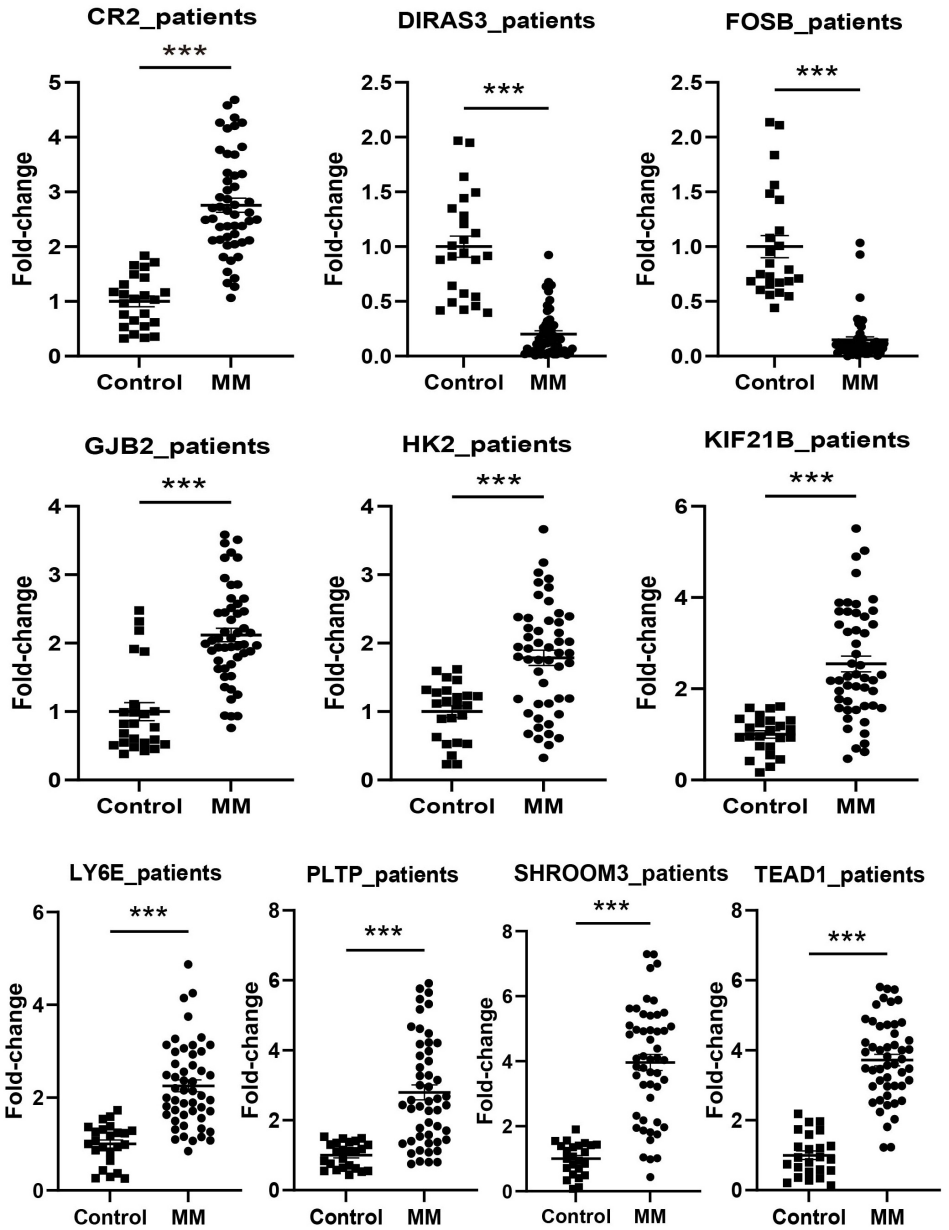
Experimental validation in patients (Control: n = 24, MM: n = 50). ***P < 0.001.

The expression levels of *CR2*, *GJB2*, *HK2*, *KIF21B*, *LY6E*, *PLTP*, *SHROOM3*, and *TEAD1* were upregulated in all cell lines used in the experiments (RPMI8226, MM1.R, U266, NCI-H929, and LP-1) (P < 0.05), in contrast to *FOSB* (P < 0.001). *DIRAS3* was downregulated in MM1.R, U266, and LP-1 (P < 0.001) (**Figure 11**). We next performed further validation in collected clinical BM specimens. Consistently, *DIRAS3* and *FOSB* showed a trend of downregulation in MM patients relative to normal subjects (P < 0.001), whereas the other genes showed the opposite trend (P < 0.001) (**Figure 12**).

## 4 Discussion

The development of biomarkers for MM is rapidly evolving, driven by new technologies, based on a deeper understanding of the biological mechanisms of tumors, with the goal of personalized patient management. Metabolic reprogramming is the key feature of tumors, which frequently causes tumor cells to become highly dependent on particular metabolic routes. Targeting tumor metabolism has gained widespread attention in the era of precision medicine (39).

Disulfidptosis describes a novel form of programmed cell death. Under glucose deprivation, *SLC7A11* dominates disulfide and ROS accumulation, mediating aberrant disulfide bond formation and F-actin collapse (10). Moreover, thiol oxidizers and GLUT inhibitors were shown to facilitate this process (10). The potential effects of disulfidptosis in cancers such as hepatocellular carcinoma (40), pancreatic ductal adenocarcinoma (41), glioblastoma (39), and gastric cancer (42) have been revealed. However, the specific role that disulfidptosis plays in MM is unclear.

In our study, we identified three subtypes based on DRGs. Liu et al. screened out the top three suppressor hits through CRISPR-Cas9: *SLC7A11*, *SLC3A2* and *RPN1* (10). These three genes play a key role in disulfidptosis. They were expressed at higher levels in C1 than in C2 and C3. The expression of *SLC3A2* in C2 was higher than that in C3. Additionally, compared with C3, the upregulated pathways in C2 included ROS metabolism and actin binding, etc. All these indicate that C1 and C2 are more susceptible to the perturbation of disulfidptosis. Survival analysis showed that C2 had a worse survival outcome than C3. Such prognostic differences may be due to the enrichment of C2 in pathways such as HIF-1 signaling pathway and autophagy, its resistance to doxorubicin and lenalidomide, and its higher likelihood of T-cell dysfunction and rejection. Meanwhile, C1 has the lowest immune score and the highest level of T-cell exclusion. C1 and C2 were more likely to undergo immune escape, suggesting a poorer response to immunotherapy. However, in the future, GLUT inhibitors that promote disulfidptosis may be an effective therapeutic strategy for targeting C1 and C2.

MM, characterized by high glucose consumption, has been found to rely on multiple glucose transporters for survival (43). Multiple inhibitors can induce apoptosis and autophagy in MM cells through glucose deprivation, and reduce resistance to traditional chemotherapeutic agents such as melphalan and bortezomib (44, 45). A high synthesis rate of disulfide bond-rich proteins is another feature of MM, depending on redox balance for proper protein folding (46, 47). The upregulated Trx system in various tumors, including MM, provides a platform for clearing ROS interference in malignant plasma cells (14, 48, 49). The high expression of PDI in relapsed and refractory MM endows them with precise regulation of protein folding and further resistance to proteasome inhibitors (PIs) (17, 18, 50). Not surprisingly, both Trx inhibitors and PDI inhibitors exhibited anti-MM efficacy *in vitro* and *in vivo*, even against PI-resistant cells (14, 51–53). Actin-related genes are considered candidate driver genes for MM (54–56). Actin polymerization can mediate the homing of MM cells to the BM ecological niche and their interactions with protective components of the microenvironment, which is strongly associated with the pathogenesis, invasiveness, and drug resistance of MM (57, 58). Furthermore, several DRGs have been found to play important roles in MM. *SLC3A2*, which acts as an amino acid exchanger, is one of the most abundant proteins on the surface of myeloma cells (59), providing support for the secretion of copious amounts of antibodies by MM cells (60). Its knockdown prevents B cells from proliferating and differentiating into plasma cells (61). Moreover, *SLC3A2* has been shown to drive mTORC1 activity in MM to increase invasiveness (62) and is a target for immunomodulatory drug activity (63). The phosphorylation of *RPN1* contributes to the correct assembly of the 26S proteasome. Phosphorylation blockade results in cell growth inhibition and mitochondrial dysfunction (64). *RPN1* is aberrantly activated in MM and its mediation of aberrant endoplasmic reticulum autophagy may be tied to the genesis and development of MM (65, 66). *RAC1* belongs to the Ras proto-oncogene superfamily and regulates cell proliferation, cytoskeletal reorganization, and cell migration (67, 68). *RAC1* is involved in the adhesion of myeloma cells in the BM, which contributes to drug resistance and invasiveness (57, 69). Next, we constructed a prognostic model based on DEGs between subtypes. In both the training and validation sets, the high-scoring group showed a worse prognosis. Similarly, the biological differences behind the distinct subgroups were mainly enriched in oxidative phosphorylation, proteasome, p53, PI3K-AKT, and JAK-STAT signaling pathways. Several studies have revealed the associations between these pathways and the pathogenesis of MM (70–76). In addition, we observed that T cells in the high-scoring subgroup microenvironment were more susceptible to rejection and had a higher likelihood of relapse.

Moreover, we performed a drug target analysis. Some predicted drugs targeting the high-risk group have already been studied in myeloma. There is evidence that paclitaxel has anti-MM stem cell action (77), and synergistically interferes with mitosis and induces apoptosis with other drugs such as dexamethasone (78, 79). In a phase II study of relapsed refractory MM, paclitaxel showed certain clinical benefits (77). Polo-like kinase inhibitor BI-2536 is a cell cycle regulator and its killing effect has been validated in MM cells and xenograft models (80, 81). The VAD regimen (vincristine, doxorubicin, and dexamethasone) is widely recognized as the standard initial induction regimen for MM. It induces early tumor load reduction and less toxicity to BM stem cells (82). Leptomycin B, an inhibitor of nuclear translocation with potent antitumor effects, was found to increase the sensitivity of myeloma cells to doxorubicin (83). The output protein inhibitors have shown the effect of inducing apoptosis in MM cells, inhibiting osteolysis, and improving survival (84, 85), and several clinical trials are underway (84). As a KSP inhibitor, SB-743921 was discovered the function of inducing MM cell death by blocking the NF-κB signaling pathway (86). It is presently being researched in clinical trials related to myeloma, leukemia, and solid tumors (87, 88). Drug-resistant MM cells have reportedly been effectively targeted by gemcitabine through its inhibition of DNA damage repair (89–91). The autologous stem-cell transplant regimen consisting of gemcitabine, busulfan, and melphalan demonstrated greater efficacy than a high-dose melphalan regimen (92). Rigosertib has been identified in both CTRP and PRISM, suggesting its great potential as a new therapeutic tool for MM. Rigosertib is a multi-kinase inhibitor, showing high potency against a wide range of tumors both *in vivo* and *in vitro* (93). It is being evaluated in several clinical trials for refractory B-cell malignancies (e.g., MM and chronic lymphocytic leukemia) (94). Although some of the predicted drugs are not standard agents in MM treatment, the above studies show their antitumor effects in MM. Our future studies will further explore the potential of these drugs in combination therapy and specific subtype-targeted therapies through *in vivo* and *in vitro* experiments.

The miRNA/TF-mRNA network was further constructed to clarify the regulatory interactions of the candidate genes. miR-193 inhibition was found to induce overexpression of the anti-regulatory protein MCL-1 in MM (95). As a well-known oncogenic cluster (96, 97), by blocking p53 and turning on the PI3K/AKT pathway, miR-25 promotes MM proliferation (98, 99). Upregulation of miR-365 was reported to inhibit myeloma cell proliferation (100). Among TFs, bromodomain-containing (BRD)-4 is a promising therapeutic target for regulating the expression of oncogenes such as *MYC* in multiple cancers, including MM (101). Consistent with previous drug prediction analyses, bromodomain inhibitor OTX015 has great therapeutic potential in MM with the ability to modulate NF-κB, cell cycle, EGFR, and proliferative signaling pathways (102). It shows strong antiproliferative properties *in vitro* MM assays, promotes osteoblast differentiation and inhibits osteoclast activity *in vivo* (102). Furthermore, phase I trials demonstrated the favorable anti-MM activity and safety profile of OTX015 (103, 104). Another TF, EP300, is one of the most frequently altered genes in MM chromatin regulators (105). Inhibition of EP300 BRDs leads to apoptosis, cycle arrest, and synergistic enhancement of NK cell-mediated cytotoxicity through inhibition of *IRF4* and *MYC* (106, 107). A related inhibitor, CCS1477, is also being assessed in a clinical trial (NCT04068597, 2019-08-09) that includes MM.

In the subsequent prediction of 1-, 3-, and 5-year survival, the nomogram combining age, ISS, and risk score demonstrated higher accuracy than R-ISS and ISS in both the training and validation datasets. This reflects the potential feasibility of using disulfidptosis-related scores for survival prediction in clinical practice.

The genes that make up the prognostic model are also closely related to MM. Overexpressed in many malignancies, *HK2* is a crucial enzyme that catalyzes the initial stage of glycolysis (108–110). Additionally, aberrant activation of *HK2* is linked to poor outcomes and PI resistance in myeloma patients (111, 112). Targeting *HK2* has emerged as a promising treatment for myeloma (113, 114). As a crucial component of the Hippo signaling pathway, *TEAD1* regulates cell division, proliferation, and death (115). The cytotoxicity of carfilzomib was found to be related to a decrease in *TEAD1* expression (116). *KIF21B*, belonging to the kinesin family, participates in the intracellular transport of membrane organelles. Its overexpression is an important feature of high-risk MM (117). Extensively and abundantly expressed in malignancies, *LY6E* is a GPI-anchored cell surface protein that controls T lymphocyte activation (118). It is crucial for TGF-β, PI3K/Akt signaling pathway, and HIF-1 transcription (119), and is closely associated with the progression, immune escape, stem cell-like features, and drug resistance of multiple cancers (118, 120). Currently, anti-*LY6E* antibodies have shown antitumor activity and acceptable safety in phase I trials of refractory malignant tumors (NCT02092792) (121). *GJB2* has been found a supportive role as a junction protein in acute myeloid leukemia, potentially linked to development and chemotherapy sensitivity (122, 123). Encoding lipid transfer proteins, *PLTP* is important in tumor growth (124–126). The type 2 complement receptor (*CR2*) has recently been redefined as an inhibitory co-receptor that mediates the inhibition of human B lymphocyte function, including the release of cytokines and antibodies (127). *SHROOM3* is crucial for regulating cytoskeletal proteins and has been identified as a novel coding variant in high-risk neuroblastoma (128). *DIRAS3*, which encodes a tumor suppressor factor, can mediate the inhibition of cell growth and malignant transformation (129). Furthermore, MM endothelial cells exhibit suppressed *DIRAS3* expression, which may be related to their high-risk excessive angiogenesis phenotype (130). *FOSB* is considered a regulatory factor for cell proliferation, differentiation, and transformation. Research has demonstrated that the BM microenvironment can assist MM cell survival by inhibiting *FOSB* (131).

Our study still has several limitations. Firstly, additional validation of our model in a larger multicenter population is required. The prognostic value of the model in our clinical samples needs to be evaluated in subsequent studies. Second, given the limited number of MM datasets in public databases, the selected validation sets lacked important clinical information such as R-ISS. Future studies should focus on multicenter data integration and exploration of new prognostic markers to further enhance the clinical value of the model. Finally, it is necessary to improve experiments based on more samples and mechanisms in the future.

## Conclusion

Our investigation explored the heterogeneity of MM by identifying subgroups with different prognoses based on disulfidptosis. The disulfidptosis-related feature is significant for predicting the survival and treatment responsiveness of MM. Disulfidptosis is expected to become a new tool of risk stratification and personalized targeted therapy for MM.

## Data Availability

The data presented in this study are deposited in the GEO database repository, with accession numbers GSE136337, GSE24080 and GSE4204.
