# Supplementary material for: Leveraging a disulfidptosis-based signature to characterize heterogeneity and optimize treatment in multiple myeloma
Source: Front Immunol. 2025 Apr 16;16:1559317. doi: 10.3389/fimmu.2025.1559317 (PMC12041008; doi:10.3389/fimmu.2025.1559317)
Supplement: Supplementary file 1 [file Table1.docx]

Supplementary Material


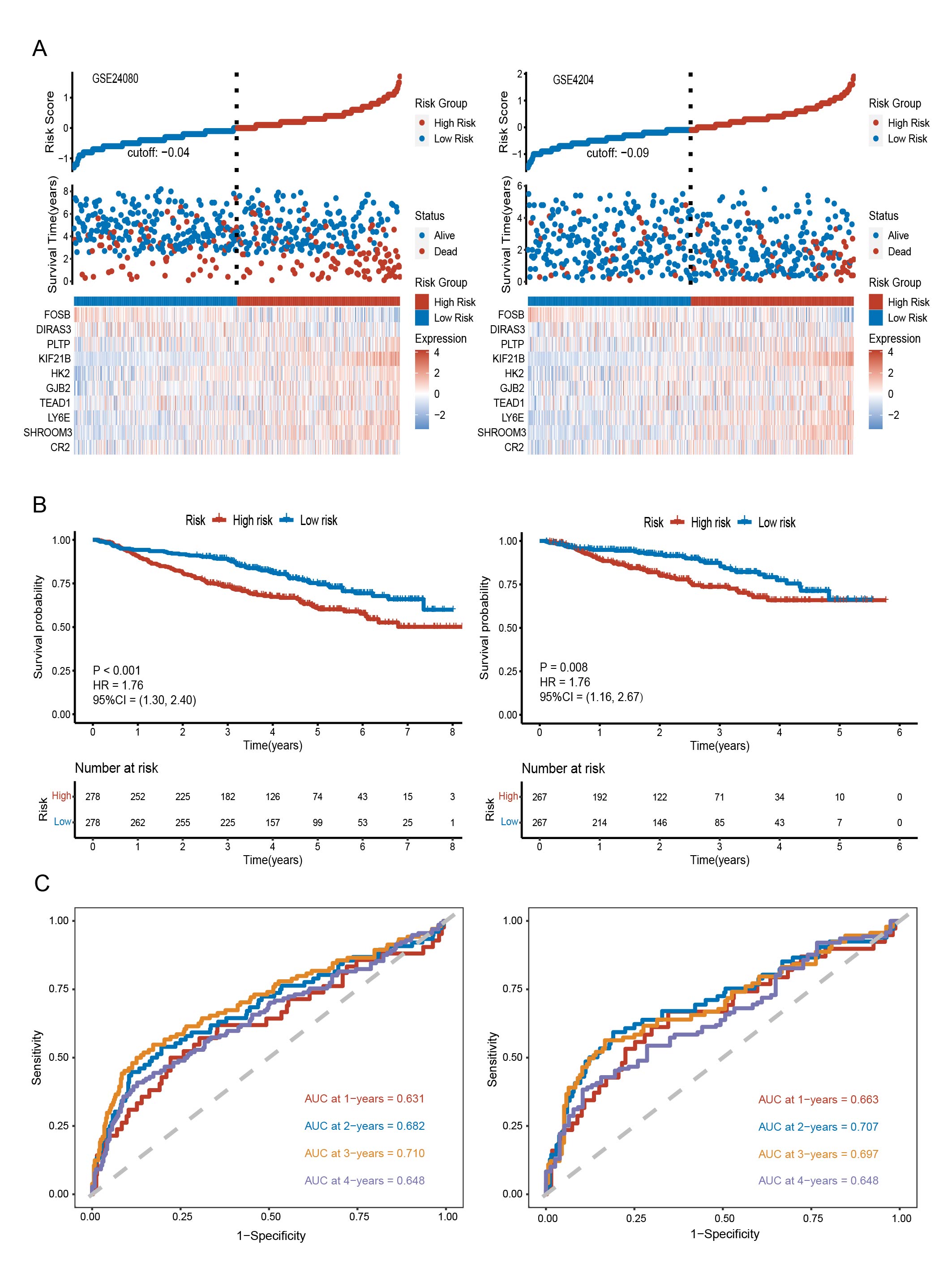


**Supplementary Figure 1.** **Validation of the prognostic model.** **(A)** Distributions of survival status between high and low-risk groups and gene expression heatmaps in the prognostic model. **(B)** Kaplan-Meier curves of high- and low-risk groups (GSE24080: P < 0.001; GSE4204: P = 0.008). **(C)** The sensitivity and specificity of the model were assessed by time-ROC analysis.
